# Maladie de Kikuchi-Fujimoto: à propos d’un cas

**DOI:** 10.11604/pamj.2017.27.144.12102

**Published:** 2017-06-28

**Authors:** Sanaa Elfihri, Mustapha Laine, Fouad Kettani, Jaouda Ben amor, Jamel Eddine Bourkadi

**Affiliations:** 1 Service de Pneumo-Phtisiologie, Hôpital Moulay Youssef, CHU Ibn Sina, Rabat, Maroc; 2 Laboratoire d’Anatomie Pathologique Nations Unies, Rabat, Maroc

**Keywords:** Maladie de Kikuchi-Fujimoto, lymphadénite histiocytaire nécrosante, adénopathies, Kikuchi-Fujimoto disease, histiocytic necrotizing lymphadenitis, adenopathies

## Abstract

La maladie de Kikuchi-Fujimoto ou lymphadénite histyocytaire nécrosante constitue une cause rare et bénigne d’adénopathies cervicales. C’est une entité anatomoclinique d’étiologie inconnue. La confirmation du diagnostic est toujours apportée par l’étude histologique ganglionnaire. L’évolution est généralement favorable avec guérison spontanée au bout de quelques semaines. Nous rapportons l’observation d’une fille de 9ans ayant consultée pour des adénopathies cervicales associées à une fièvre. La biopsie ganglionnaire cervicale a conclu à une maladie de Kikuchi. L’évolution était marquée par la régression des adénopathies sans aucun traitement.

## Abstract (en)

Kikuchi-Fujimoto disease or histiocytic necrotizing lymphadenitis is a rare cause of benign cervical adenopathies. It is an anatomoclinic entity of unknown cause. Diagnosis is based on histologic examination of the lymph nodes. Patient’s evolution is generally favorable with spontaneous healing after a few weeks. We here report the case of a 9-year old girl presenting with cervical lymphadenopathy associated with fever. Cervical lymph node biopsy showed Kikuchi-Fujimoto disease. Patient’s evolution was marked by regression of adenopathies without receiving any treatment.

## Introduction

La maladie de Kikuchi-Fujimoto (KF) ou lymphadénite histiocytaire nécrosante est une affection ganglionnaire bénigne, décrite pour la première fois en 1972 au japon par Kikuchi et Fujimoto. Elle touche essentiellement les femmes jeunes asiatiques, elle est rare et peu connue en pédiatrie. Le motif commun de consultation chez l’enfant est les polyadénopathies. C’est une maladie d’étiologie indéterminée, une origine auto-immune est probable. Le tableau clinique et biologique est peu spécifique et seul l’examen anatomopathologique d’un ganglion permet le diagnostic. Nous rapportons le cas d’un enfant de 9 ans suivi pour des polyadénopathies cervicales inflammatoires chroniques révélatrices de la maladie de Kikuchi.

## Patient et observation

Une fille âgée de 9 ans, suivie pour polyadénopathies cervicales unilatérales gauche dans un contexte fébrile et amaigrissement non chiffré évoluant depuis un mois. Elle n’avait pas d’antécédents personnels ou familiaux particuliers ni de contage tuberculeux. Elle était asymptomatique sur le plan respiratoire. L’examen clinique avait montré plusieurs adénopathies jugulo-carotidiennes et spinales gauches dont la plus volumineuse mesurait presque 2cm, de consistance ferme, douloureuses et mobiles. Il n’y avait pas de porte d’entrée infectieuse. La température chiffrée à 39°C. Le bilan biologique avait montré une hyperleucocytose à 7400 à prédominance neutrophiles, une CRP à 3,5, une VS à 40 mm à la première heure et la recherche de BK dans les expectorations était négative à l’examen direct et à la culture. L’intradermoréaction à la tuberculine était positive à 12mm. Les sérologies de l’hépatite B, C et HIV étaient négatives, l’examen cytobactériologique des urines était négatif.

**Figure 1:**
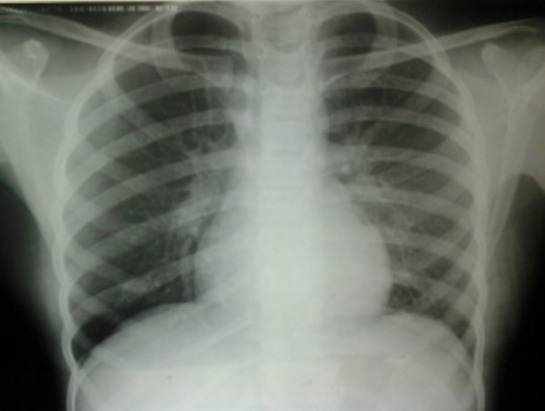
Radiographie thoracique: opacités hilaires

**Figure 2:**
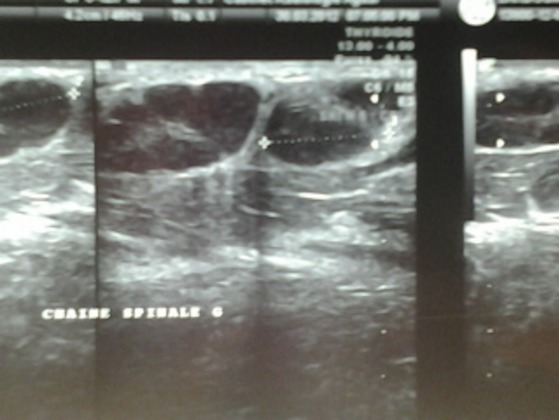
Echostructures ovoïdes hypoéchogènes

**Figure 3:**
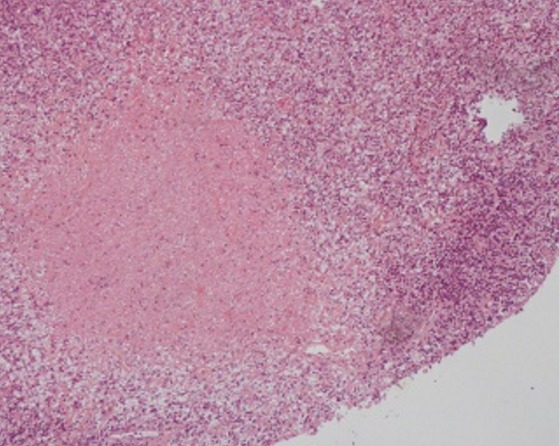
Foyers de nécrose bordés par des histiocytes et de cellules immunoblastiques. HES Gx100

**Figure 4:**
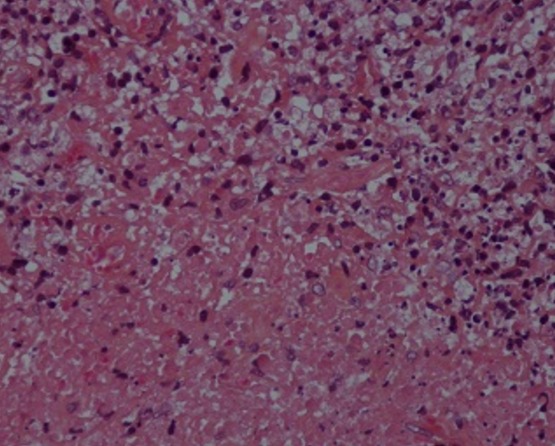
HES Gx400

**Figure 5:**
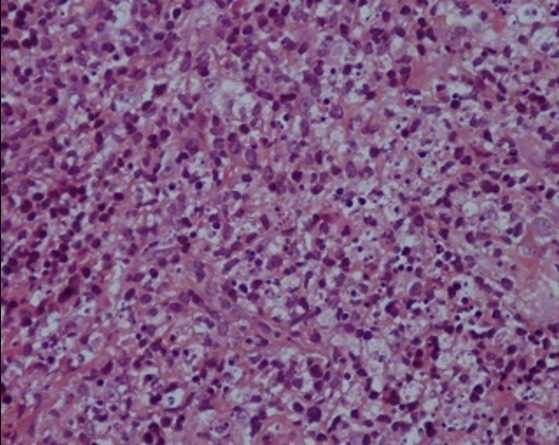
Foyers de nécrose bordés par des histiocytes spumeux et de cellules immunoblastiques atypiques. HES Gx400

La radiographie thoracique avait montré des opacités hilaires bilatérales (Figure 1). La tomodensitométrie thoracique avait objectivé des adénopathies médiastinales. L’échographie cervicale avait mis en évidence de nombreuses adénomégalies des chaines jugulocarotidiennes et spinales gauches avec un diamètre de 6- 15mm, beaucoup plus nombreuses au niveau de la chaine spinale gauche, les plans musculaires sont respectés avec absence d’anomalies de la peau et de la graisse sous cutanée (Figure 2). L’examen anatomopathologique était pathognomonique de lymphadénite nécrosante de KiKuchi: une architecture normale et globalement conservée avec présence de nombreux foyers de nécrose plus ou moins étendus bordés parfois par des histiocytes spumeux accompagnés de cellules immunoblastiques légèrement atypiques, sans polynucléaires neutrophiles ni de plasmocytes (Figure 3, Figure 4 et Figure 5). La conduite thérapeutique s’est limitée à une surveillance. L’évolution était marquée par une amélioration clinique et radiologique sans récidive avec un recul de 4 ans.

## Discussion

La maladie de KF est définie comme une lymphadénite histiocytaire nécrosante de cause inexpliquée [1, 2]. Elle a été décrite pour la première fois en 1972 au japon par deux anatomopathologistes KIKUCHI et FUJIMOTO [3, 4], elle est révélée; comme dans notre observation; par des adénopathies fébriles généralement cervicales fermes, volumineuses parfois douloureuses mais ne s’ulcèrent jamais. C’est une affection rare chez l’enfant dont la connaissance est importante permettant d’éviter des investigations complémentaires souvent inutiles et parfois invasives. La durée moyenne entre l’apparition des premiers signes chez l’enfant et l’établissement du diagnostic est estimée à 3 semaines (6 semaines pour notre observation). Elle Concerne essentiellement les garçons : sexe ratio 1,9/1 contre un sexe ratio ¼ à l’âge adulte et touche préférentiellement les enfants d’origine africaine. L’âge moyen de survenue est de 8 ans [5]. Le tableau clinique est dominé par la constance des adénopathies qui sont souvent unilatérales localisées en région cervicale postérieure, tel le cas pour notre patiente [6, 7]. Des localisations moins fréquentes ont été décrites, superficielles au niveau des chaines épi-trochléennes, sus claviculaires, inguinale ou profondes au niveau coelio-mésentérique ou médiastinales [8]. Pour notre cas, une TDM thoracique a mis en évidence une localisation médiastinale. La fièvre n’est pas constante, présente dans 37,4% des cas.

Les signes cliniques associés sont les lésions cutanées dans 16 à 40% des cas : urticaire morbiliforme mais peut également s’apparenter à des lésions non spécifique de lupus, un tableau d’appendicite peut être simulé par une adénite mésentérique, une hépatosplénomégalie, des arthralgies, des sueurs nocturnes, un syndrome méningé, des atteintes myocardique et médullaires ont été décrites [9]. Dans notre observation, il n’y avait pas de signes associés. Les perturbations biologiques sont peu spécifiques: la vitesse de sédimentation est souvent élevée, une leucopénie, parfois une lymphocytose, rarement une anémie ou une thrombopénie, le bilan hépatique peut être perturbé à type de cytolyse ou cholestase [10, 11]. La confirmation diagnostique de cette affection repose sur l’examen anatomopathologique d’une biopsie ganglionnaire. Une architecture ganglionnaire partiellement conservée avec hyperplasie folliculaire, des zones de nécrose dans les régions corticales et paracorticales caractérisées par des plages cellulaires mélant des histyocytes (CD 68+) dont des cellules plasmocytoides et de grandes cellules lymphoides T CD8 en transformation immunoblastique. L’absence de polynucléaires neutrophiles et d’éosinophiles est un signe négatif constant. Trois formes histologiques sont décrites: la forme nécrosante la plus fréquente (cas de notre observation), la forme proliférative pseudo tumorale et la forme xanthomateuse [12]. L’étiopathogénie demeure inconnue, les deux hypothèses soulevées sont infectieuses et auto-immune.

L’origine infectieuse est suspectée devant l’association fréquente à une infection virale, bactérienne ou parasitaire. L’hypothèse auto-immune semble en cause par la forte association de la maladie de KF au lupus érythémateux disséminé, des cas de lupus ayant précédé, révélé ou a fait suite à une maladie de KF ont été décrits. Les principaux diagnostics différentiels sont les lymphomes, la tuberculose ganglionnaires, le lupus systémique, la toxoplasmose, l’infection VIH et la maladie de Kawasaki. L’évolution spontanée est généralement bénigne avec guérison sans séquelles en quelques semaines à quelques mois. Ce fut le cas pour notre patiente avec une régression des adénopathies et disparition des signes généraux un mois après le diagnostic. L’intensité des signes cliniques peut parfois nécessiter une corticothérapie orale ou en bolus. L’exérèse chirurgicale ganglionnaire semblerait accélérer la guérison [5]. Des cas de décès ainsi que des cas de récidives après quelques années ont été décrites nécessitant à chaque fois une confirmation histologique. La fréquence moyenne de récidive est de 3,7%, elle survient habituellement au niveau du site initial de la maladie [13]. Un suivi régulier et à long terme est essentiel afin de dépister une récidive ou un éventuel lupus [14]. Après un recul de 4 ans, notre patiente n’a pas présenté de récidive.

## Conclusion

La maladie de KF est rare chez l’enfant. Sa connaissance est intéressante car elle permet de limiter les examens complémentaires notamment invasifs. Elle doit être évoquée après les causes infectieuses et tumorales devant les adénopathies d’origine indéterminée. L’examen anatomopathologique de l’adénopathie peut confirmer le diagnostic. La guérison est constante mais un suivi régulier est recommandé du fait de la possibilité d’évolution vers un lupus érythémateux disséminé.

## Conflits d’intérêts

Les auteurs ne déclarent aucun conflit d’intérêt.
